# Joint Temperature-Lasing Mode Compensation for Time-of-Flight LiDAR Sensors

**DOI:** 10.3390/s151229854

**Published:** 2015-12-11

**Authors:** Anas Alhashimi, Damiano Varagnolo, Thomas Gustafsson

**Affiliations:** Control Engineering Group, Department of Computer Science, Electrical and Space Engineering, Luleå University of Technology, Luleå 97187, Sweden; damiano.varagnolo@ltu.se (D.V.); thomas.Gustafsson@ltu.se (T.G.)

**Keywords:** LiDAR, EM, Gaussian mixture model (GMM), mode hopping, ToF, maximum likelihood (ML), multi-modality, SICK LMS200

## Abstract

We propose an expectation maximization (EM) strategy for improving the precision of time of flight (ToF) light detection and ranging (LiDAR) scanners. The novel algorithm statistically accounts not only for the bias induced by temperature changes in the laser diode, but also for the multi-modality of the measurement noises that is induced by mode-hopping effects. Instrumental to the proposed EM algorithm, we also describe a general thermal dynamics model that can be learned either from just input-output data or from a combination of simple temperature experiments and information from the laser’s datasheet. We test the strategy on a SICK LMS 200 device and improve its average absolute error by a factor of three.

## 1. Introduction

ToF LiDARs estimate distances by emitting short bursts of laser light and by measuring the time it takes for the reflected photons to arrive back to the device [1]. Despite being based on a very simple principle, they are very much both accurate and precise devices [2]: for example, precisions can reach 10 mm of standard error when the object is 10 m away. Due to these favorable properties, they are commonly used in critical industrial applications where there is the need for high quality measurements.

It is well known that these devices need temperature compensation mechanisms, since changing their temperature leads to changes in the statistics of the returned measurements. The effect of temperature may be huge: experiments by [3] on an amplitude-modulated continuous-wave laser radar pointing at a target six meters away from the sensor showed that measurements at 21 °C and measurements at 45 °C were differing by 40 cm. Since thermal stabilization of a laser scanner may take up to 30 min [4], it is clear that these sensors are affected by a warm-up-induced time drift that must be compensated. Manufacturers of ToF devices thus usually embed opportune algorithms in their products that implement this temperature compensation mechanism.

Unfortunately, temperature is not the only physical factor that deserves compensation: as described in detail in Section 2, lasers can suddenly change their lasing mode. This property, called the mode-hopping effect, has a substantial impact on the measure returned by ToF devices, since changing lasing mode means to change the spectral content of the laser burst, *i.e.*, change its time of flight. Remarkably, to the best of our knowledge, the existing literature does not focus on managing this effect, but rather, considers only temperature compensation mechanisms.

We would like to mention here that there are also other methods with high temperature compensation at the nano-scale measurement, such as [5]. The method reduces the offset, the temperature characteristic of the main sensing element, the temperature drift and the noise by the switching method.

### 1.1. Statement of Contributions

We propose an expectation maximization (EM) algorithm that compensates mode-hopping effects by modeling the induced measurement noise as a Gaussian mixture. Thus, from the mathematical perspective, we introduce some latent variables (namely, from which Gaussian the noise comes from) as additional estimands. This EM algorithm is also coupled to a temperature compensation filter that is built on a physics-based linear model for the thermal dynamics of the laser scanner. Summarizing, thus, our contributions are, with respect to the aforementioned literature:

1. A thorough motivation for why it is meaningful to consider mode-hopping effects in laser scanners, arising from a physical description of the lasing mechanism in laser diodes;
2. A thermodynamical model for the thermal dynamics of a whole laser scanner, needed by the proposed strategy to account for temperature effects;
3. A statistical model describing the measurement process that decouples the effects of the mode-hopping from temperature effects;
4. A numerically-efficient EM strategy based on the statistical model above;
5. A validation of the proposed compensation strategy on real devices.

With the validation, we also show that it is possible to improve the absolute error of a SICK LMS 200 device by a factor of three.

### 1.2. Organization of the Manuscript

Section 2 analyzes the effects of the laser temperature on the measured distance. Section 3 proposes a general model for the thermal dynamics of a pulsed ToF LiDAR. Section 4 presents a general measurement model accounting for both temperature and mode-hopping effects. Section 5 describes how to train the EM algorithm, while Section 6 describes how to use the same algorithm for testing purposes. Section 7 then proposes a likelihood ratio test for calibrating the hyperparameters of the EM algorithm. Section 8 presents some numerical results on commercial devices. Finally, Section 9 draws some conclusions and future research directions.

## 2. Effects of the Laser Temperature on the Measured Distance

This section lays down interpretations motivating the structure of the novel compensation procedures. We thus here describe the functioning principle of ToF scanners, explain why the measured distance depends on the temperature of the device and motivate why the measurement noise of a LiDAR is intrinsically multi-modal.

**Figure 1:**
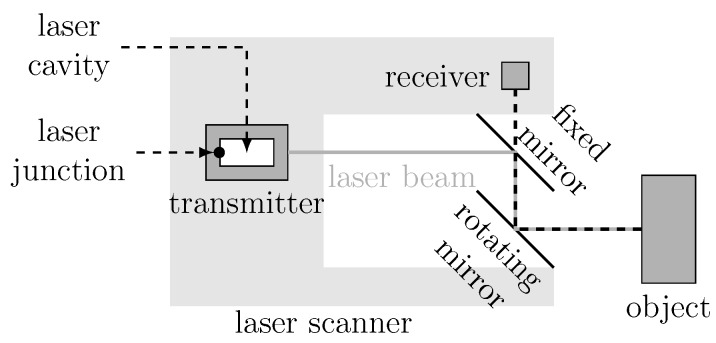
Graphical description of the operating principle for pulsed ToF LiDARs. A pulsed infra-red laser beam is first emitted from the transmitter. The case of the transmitter, in dark gray, encloses a laser junction and a laser cavity. The emitted laser beam is then deflected by a rotating mirror (resulting in a fan-shaped scan pattern) and, finally, reflected from the object surface. The time of flight *τ* between the transmission and the reception of the laser beam is then used to estimate the distance *d* between the scanner and the object.

Consider then the basic operation of pulsed ToF LiDARs in Figure 1 and in its caption.

The measurement of the distance derives from ideal considerations: if the temporal width of the pulse is null, then the distance *d* between the sensor and the object should satisfy:

(1) $$d=\frac{c\;\tau }{2}$$

where *c* is the speed of light and *τ* is the measured ToF between when the laser pulse is emitted and when it is received. Assume ideally that the laser pulse contains photons with a unique nominal wavelength $\lambda_{0}$. Since:

(2) $$c=\lambda_{0}f$$

with *f* the light frequency and $\lambda_{0}$ the nominal wavelength, and since the light frequency *f* remains the same through different media, to know *c*, it is sufficient to know $\lambda_{0}$. Thus, from knowing $\lambda_{0}$, one can compute *d*, since *τ* is measured.

We can already now notice the first effect of the temperature of the device on the measurement: according to its datasheet, the nominal wavelength of the laser diode SPL PL90 from OSRAM [6] is 905 nm at 25 °C, and 907.8 nm at 35 °C. Assuming the target to be at a one-meter distance, this 2.8 nm variation in the wavelength *λ* then results in a 10.3-ps variation in the ToF *τ*, *i.e.*, a variation in the measured distance of approximately 3.1 mm.

**Figure 2:**
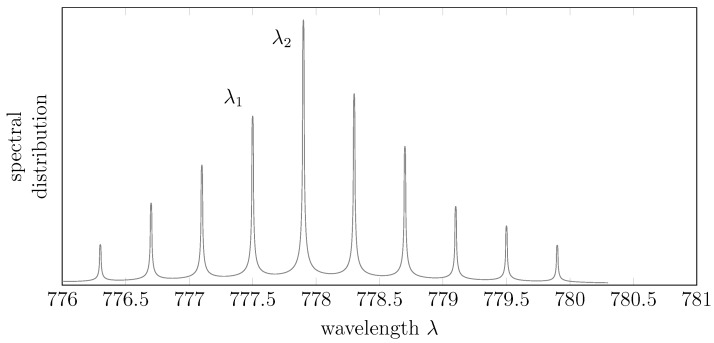
Average spectral distribution for GaAsP lasers at the nominal temperature of 21 °C [7].

**Figure 3:**
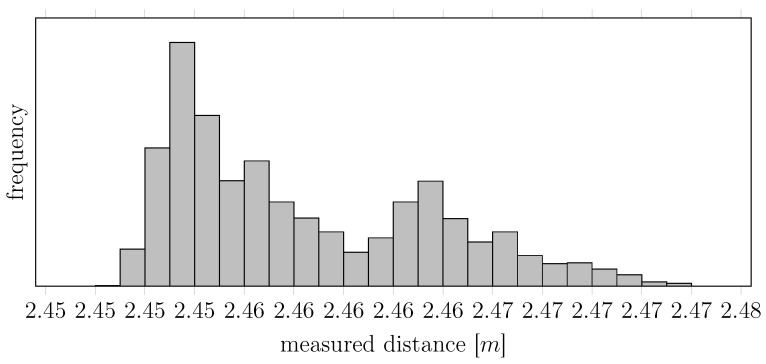
Histogram of consecutive measurements returned by a SICK 200 device in thermodynamical and electrical equilibrium pointing at a fixed object and in a controlled environment.

**Figure 4:**
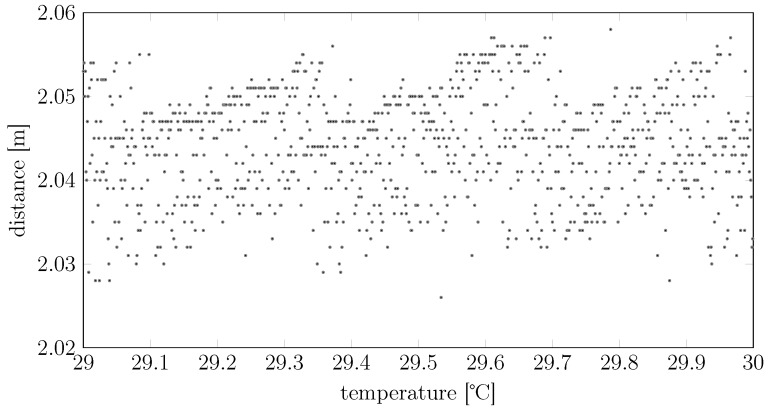
Dependency of the distance measurements on the device temperature for a SICK 200 device pointing to a fixed object in a controlled environment. Despite the true distance and other parameters potentially affecting the measurements being constant in time, the distributions of the measurements are temperature varying. We can notice how the device compensates for the temperature change by adding a temperature-varying negative bias, but that it does not compensate the mode-hopping effect.

The previous consideration is nonetheless simplistic. We can indeed notice another three distinct effects:

1. In general, lasers do not emit at a unique frequency *λ*. Indeed, the average spectral distribution of the laser pulses follows a “comb”-like density, like the one in Figure 2.
2. Lasers are affected by the so-called mode-hopping effect [8] and, indeed, oscillate between different lasing modes (the teeth of the “comb” of Figure 2), for which two different pulses generated under the same temperature and external conditions may have different *λ*’s (e.g., referring to the same figure, the first pulse may contain only photons with wavelengths $\lambda_{1}$, while the second pulse may contain only photons with wavelengths $\lambda_{2}$). In other words, the actual distribution of one specific pulse may contain only a subset of the teeth of the average spectral distribution. Using naively Equations (1) and (2) to estimate *d* without being aware of the mode hopping, *i.e.*, assuming a certain $\lambda_{0}$ without actually knowing that the average *λ* jumps between different lasing modes, reflects thus in a multimodal measurement of *d*, as clearly shown in Figure 3.
3. The average spectral distribution of the laser pulses is not fixed, but rather depends on the temperature of the transmitter [9]. More precisely, the positions and amplitudes of the modes in Figure 2 depend on both the current flowing through the laser junction and the geometry of the laser cavity, but eventually, these two effects are inter-combined: the current flow produces heat that will modify the geometry of the cavity. Eventually, thus, the temperature affects the position and amplitude of the modes of the average spectral distribution. This temperature effect can be clearly seen in Figure 4: even if the device is nominally already compensated in temperature, one can clearly see two different lasing modes shifting in temperature.

To summarize, the actual *λ* of a laser pulse is in general different from the nominal $\lambda_{0}$ because of two effects: first, the laser may oscillate between different modes; second, these positions of these modes vary with temperature.

## 3. A General Model for the Thermal Dynamics of a Pulsed ToF LiDAR

Instrumental for compensating the effects of temperature offsets highlighted in the previous section, we here discuss a general thermodynamic model describing the temperature dynamics of the whole laser scanner that will lead us to being able to represent the temperature changes of the laser cavity.

### 3.1. Physical Modeling

Just like any power electronic device, LiDARs generate heat that is then exchanged with the environment, so that the temperature of a scanner depends on the temperature of the environment. The main sources of heat inside the LiDAR are thus the laser diode, the motor and the electronic components of the system. The heat generated inside the scanner is then transferred to the surrounding environment through the case. Since our experience indicates that motors and other electronic components induce negligible thermal effects, we consider only the heat produced by the laser diode.

**Figure 5:**
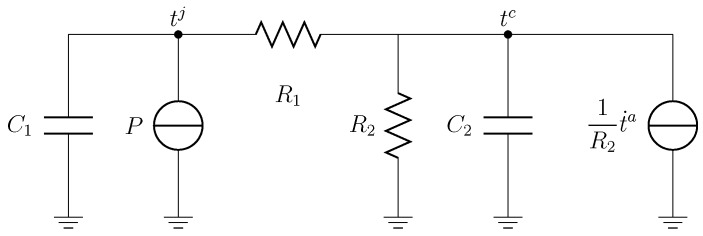
The proposed thermodynamic model for a generic pulsed ToF LiDAR.

We thus represent the thermal model of a generic scanner as the equivalent electrical circuit shown in Figure 5, interpretable as follows: when the laser is turned on, the heat generated by the laser junction is dissipated in the surrounding environment through first the transmitter case and then, second, through the laser scanner case.

Considering the notation: 


*P*
Heat power generated by the junction (equal to zero when the device is off)
$t^{j}$
Temperature of the junction
$t^{c}$
Temperature of the transmitter case
$t^{a}$
Temperature of the external ambient
$t^{s}$
Noisy measurement of the temperature of the transmitter case
$C_{1}$
Thermal inertia of the transmitter case
$C_{2}$
Thermal inertia of the laser scanner case
$R_{1}$
Thermal resistance between the transmitter case and the laser scanner case
$R_{2}$
Thermal resistance between the laser scanner case and the ambient

It follows that the dynamics for the temperatures of the laser junction and scanner case when the laser is on are thus:

(3) $$\left\{\begin{array}{c} {\dot{t}}^{c}=\frac{t^{j}}{R_{1}C_{2}}-t^{c}\left(\frac{1}{R_{2}C_{2}}+\frac{1}{R_{1}C_{2}}\right)+\frac{1}{C_{2}R_{2}}{\dot{t}}^{a}\;\\ {\dot{t}}^{j}=\frac{P}{C_{1}}+\frac{t^{c}}{R_{1}C_{1}}-\frac{t^{j}}{R_{1}C_{1}} \end{array}\right.$$

Discretizing the previous dynamics with a discretization step of *T* s and letting:

(4) $${\bar{t}}_{k}:=\left[\begin{array}{c} t_{k}^{c}\\ t_{k}^{j} \end{array}\right]\;\;{\bar{u}}_{k}:=\left\{\begin{array}{cc} P & \;\text{if}\;\text{on,}\\ 0 & \;\text{if}\;\text{off,} \end{array}\right.\;\;{\bar{\nu }}_{k}:=t_{k}^{a}-t_{k-1}^{a}$$

implies the following discrete-time state-space representation of the model:

(5) $$\left\{\begin{array}{cc} {\bar{t}}_{k+1} & =A{\bar{t}}_{k}+B{\bar{u}}_{k}+B^{\prime }{\bar{\nu }}_{k}\\ t_{k}^{s} & =C{\bar{t}}_{k}+{\bar{\mu }}_{k} \end{array}\right.$$

where $t_{k}^{s}$ is a noisy temperature measurement of the case at time *k*, ${\bar{\nu }}_{k}$ and ${\bar{\mu }}_{k}$ are independent process and measurement noises, and where:

(6) $$A=\left[\begin{array}{cc} 1-\frac{T}{R_{2}C_{2}}-\frac{T}{R_{1}C_{2}} & \frac{T}{R_{1}C_{2}}\;\\ \frac{T}{R_{1}C_{1}} & 1-\frac{T}{R_{1}C_{1}} \end{array}\right]\;B=\left[\begin{array}{c} 0\;\\ \frac{T}{C_{1}} \end{array}\right]\;B^{\prime }=\left[\begin{array}{c} \frac{T}{R_{2}C_{2}}\;\\ 0 \end{array}\right]\;C=\left[\begin{array}{c} 1\;\;\;0 \end{array}\right]$$

Notice that through Equation (5), we introduce $t_{k}^{s}$, *i.e.*, a noisy measurement of the temperature of the transmitter case. This correspond to the practical assumption that perfect knowledge of the actual case temperature $t_{k}^{c}$ is in general unavailable, since temperature sensors attached to the case of the scanner will never give noiseless recordings.

### 3.2. Identifying Model Equation (5)

If one has a dataset of recorded temperatures $t_{k}^{j}$, $t_{k}^{c}$ and $t_{k}^{a}$, then one may identify Equation (5) using standard system identification approaches, e.g., a prediction error method (PEM) as in [10]. For the common case where it may be difficult to obtain direct measurements of the quantities, we propose to resort to the following general strategy that uses the datasheet of the laser scanner in conjunction with noisy case temperature measurements $t_{k}^{s}$.

**Algorithm 1** Identification of model Equation (5) starting from the datasheet of a laser scanner.
1:From the datasheet of the laser scanner infer:
its thermal resistance $R_{1}$ (directly from the datasheet);its thermal capacity $C_{1}$ (by estimating the volume of the laser diode from the datasheet and multiplying it for the heat capacity of the material, also indicated in the datasheet);2:From measuring the weight and material of the case, infer its thermal capacity $C_{2}$;3:Estimate the generated heat power *P* by measuring the electrical power absorbed by the device and multiplying this quantity by 0.5 (for the estimated efficiency of generic laser diodes [11]);4:From situations where the scanner is in thermal equilibrium, calculate $R_{2}$ by calculating the difference between the measured case temperature $t_{k}^{s}$ and the ambient temperature $t_{k}^{a}$ divided by the estimated generated heat power *P*.


### 3.3. Estimating $t^{j}$ from $t^{s}$

Assume that model Equation (5) has been identified, either from measured data or using Algorithm 1, and observe that the thermal model is observable, reachable and has stable dynamics. Due to their favorable theoretical and numerical properties, we thus devise to estimate $t_{k}^{j}$ from noisy measurements of the case temperature $t_{k}^{s}$ via Kalman smoothers/filters, as in [12], *i.e.*, to let:

(7) $$\left[{\hat{t}}_{1}^{j},\ldots ,{\hat{t}}_{K}^{j}\right]=W\left[t_{1}^{s},\ldots ,t_{K}^{s}\right]$$

for an opportune (and potentially time varying) matrix *W*. As before, notice that the filtering starts from noisy measurements of the case temperature $t_{k}^{s}$, rather than from perfect measurements $t_{k}^{c}$.

## 4. A General Measurement Model Accounting for Temperature and Mode Hopping Effects

We recall that our aim is to model the effect of the temperature of the laser junction $t_{k}^{j}$ on the measured distance *d* and understand how noisy case temperature measurements $t_{k}^{s}$ help improving the accuracy on the final estimate of *d*. To this aim, we propose the following measurement model at the generic time instant *k*:

(8) $$y_{k}=d+H\left(t_{k}^{j}\right)\theta +\left(1-\Delta_{k}\right)w_{k}^{1}+\Delta_{k}w_{k}^{2}$$

where:

- $y_{k}$ is the distance returned by the sensor;
- *d* is the true distance from the object (assumed deterministic);
- $t_{k}^{j}$ is the temperature of the laser cavity at time *k*;
- The two modes $w_{k}^{1}∼N\left(\mu_{1},\sigma_{1}^{2}\right)$ and $w_{k}^{2}∼N\left(\mu_{2},\sigma_{2}^{2}\right)$ account for a bimodal Gaussian and white additive measurement noise. The Bernoulli random variable (r.v.) $\Delta_{k}∼B\left(\pi \right)$ selects the active mode at time *k*, so that *π* reflects the relative importance of the modes. Intuitively, $\Delta_{k}$ represent which lasing mode has been active during measurement $y_{k}$. We notice that here, we consider bimodal noises (*i.e.*, only two lasing modes) just for notational simplicity. It is nonetheless immediate to generalize the subsequent findings for an *M*-modal case;
- $H\left(\cdot \right)\theta$ is a non-linear transformation of the temperature of the laser junction $t_{k}^{j}$ in a measurement bias. In the following Examples 1 and 2, we show how different $H\left(\cdot \right)$’s and ***θ***’s express different maps from the junction temperature $t_{k}^{j}$ to the measurement bias.

Notice that, induced by our experience, we let the measurement noise modes $w_{k}^{1}$ and $w_{k}^{2}$ be independent from the laser junction temperature $t_{k}^{j}$. Our experiments indeed indicated that the moments of the noises are not affected by changing temperatures (at least for a range between 0 °C and 40 °C). Notice moreover that model Equation (8) is linear in ***θ***; this restrictive assumption is nonetheless essential for building distance estimation algorithms that are numerically fast.

**Example 1 **(Polynomial model). *Let:*

(9) $$H_{k}=H\left(t_{k}^{j}\right):=\left[t_{k}^{j},\;{\left(t_{k}^{j}\right)}^{2},\;\ldots ,\;{\left(t_{k}^{j}\right)}^{N}\right]$$

*and $\theta :=\left[\theta_{1},\ldots ,\theta_{N}\right]$. Then, the generic model Equation (8) specializes into:*

(10) $$y_{k}=d+\sum_{n=1}^{N}{\left(t_{k}^{j}\right)}^{n}+\left(1-\Delta_{k}\right)w_{k}^{1}+\Delta_{k}w_{k}^{2}$$

**Figure 6:**
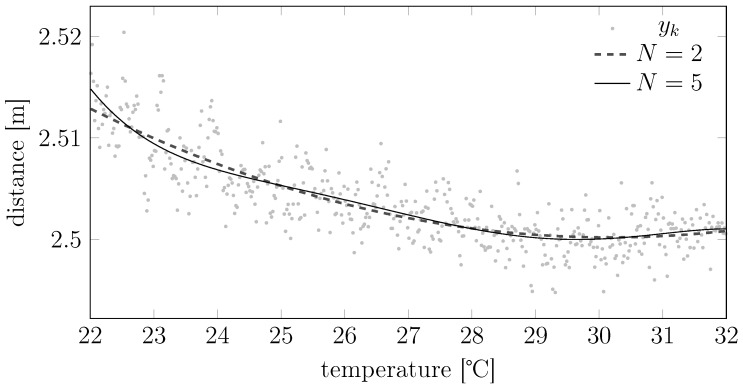
Potential temperature-bias dependencies for the polynomial model of Example 1.

*i.e., a measurement model where the temperature plays the role of an N-th order polynomial bias (see Figure 6).*

**Example 2 **(Fourier expansion model). *Let:*

(11) $$H_{k}=H\left(t_{k}^{j}\right):=\left[\cos\left(2\pi f_{0}t_{k}^{j}\right),\;\sin\left(2\pi f_{0}t_{k}^{j}\right),\;\ldots ,\;\cos\left(2N\pi f_{0}t_{k}^{j}\right),\;\sin\left(2N\pi f_{0}t_{k}^{j}\right)\right]$$

*and $\theta :=\left[\theta_{1}^{\prime },\theta_{1}^{\prime \prime },\ldots ,\theta_{N}^{\prime },\theta_{N}^{\prime \prime }\right]$, where the fundamental frequency $f_{0}$ is assumed to be known. Then, the generic model Equation (8) specializes into:*

(12) $$y_{k}=d+\sum_{n=1}^{N}\left(\theta_{n}^{\prime }\cos\left(2n\pi f_{0}t_{k}^{j}\right)+\theta_{n}^{\prime \prime }\sin\left(2n\pi f_{0}t_{k}^{j}\right)\right)+\left(1-\Delta_{k}\right)w_{k}^{1}+\Delta_{k}w_{k}^{2}$$

**Figure 7:**
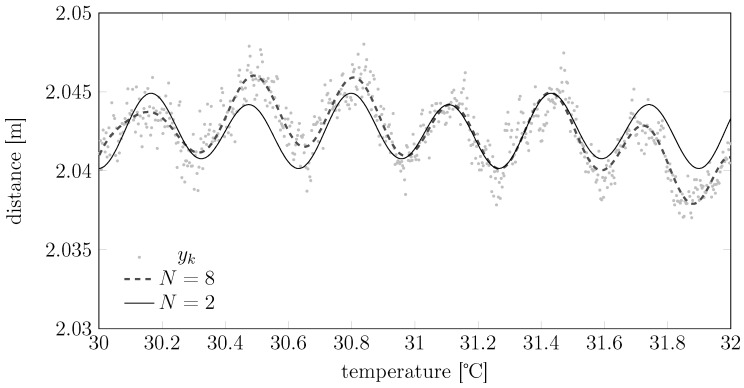
Potential temperature-bias dependencies for the Fourier expansion model of Example 2.

*i.e., a measurement model where the temperature plays the role of a bias that is periodic with frequency $f_{0}$ (see Figure 7).*

We then use model Equation (8) for practical purposes following the classical scheme:

- Design the model, *i.e.*, decide the structure for $H\left(\cdot \right)$ (e.g., between Example 1, Example 2 or also different ones depending on the need) starting from data collected in a controlled environment (see Section 7);
- Train the model, *i.e.*, estimate ***θ*** and the statistics of $w_{k}^{1}$, $w_{k}^{2}$ and $\Delta_{k}$ from data collected in a controlled environment (see Section 5);
- Test the model, *i.e.*, use the previous estimated quantities during the normal operation of the laser scanner, so as to improve the estimation of *d* from data collected in a non-controlled environment (see Section 6).

The following sections are dedicated in detail to how to implement the previous three points. Notice that Section 7, on the design of the model, is presented after Section 5 and Section 6 for notational convenience.

## 5. Training Model Equation (8)

In this section, we devise a numerical algorithm for learning ***θ*** (*i.e.*, the coefficients multiplying H(·)), $\mu_{1}$, $\mu_{2}$, $\sigma_{1}^{2}$, $\sigma_{2}^{2}$, *π* (*i.e.*, the statistics of the noises $w_{k}^{1}$, $w_{k}^{2}$ and of the random variables $\Delta_{k}$) and the values of the mode selection variables $\Delta_{k}$ starting from a dataset containing:

- $y_{k}$ for $k\in \left\{1,\ldots ,K\right\}$;
- *d*, *i.e.*, the real distance;
- $t_{k}^{s}$, *i.e.*, measurements of the temperature of the laser scanner case (to be transformed into estimates of $t_{k}^{j}$ through Equation (7)).

Let then:

(13) $$\begin{array}{ccc} y & := & {\left[\begin{array}{ccc} y_{1} & \cdots & y_{K} \end{array}\right]}^{T}\\ t^{s} & := & {\left[\begin{array}{ccc} t_{1}^{s} & \cdots & t_{K}^{s} \end{array}\right]}^{T}\\ t^{j} & := & {\left[\begin{array}{ccc} t_{1}^{j} & \cdots & t_{K}^{j} \end{array}\right]}^{T}\\ \Delta & := & {\left[\begin{array}{ccc} \Delta_{1} & \cdots & \Delta_{K} \end{array}\right]}^{T}\\ \bar{\theta } & := & {\left[\begin{array}{cccccc} \theta^{T} & \mu_{1} & \mu_{2} & \sigma_{1}^{2} & \sigma_{2}^{2} & \pi \end{array}\right]}^{T}. \end{array}$$

Given the frequentist assumptions on the unknowns, we would like to perform maximum likelihood (ML) estimation for $\bar{\theta }$, *i.e.*, seek for:

(14) $$\arg\max_{\bar{\theta },\Delta }\;P\left[y,t^{j}\;;\;d,\bar{\theta },\Delta \right]$$

Since the laser junction temperature $t^{j}$ is unavailable, Equation (14) cannot be solved. We thus resort to solving the approximated problem:

(15) $$\left(\hat{\theta },\hat{\Delta }\right):=\arg\max_{\bar{\theta },\Delta }\;P\left[y,{\hat{t}}^{j}\;;\;d,\bar{\theta },\Delta \right]$$

where ${\hat{t}}^{j}$ is the estimate of $t^{j}$ given $t^{s}$ as in Equation (7). We also notice that, instead of considering the joint density of y and ${\hat{t}}^{j}$, it is sufficient to consider the conditional density of y given ${\hat{t}}^{j}$:

**Proposition 1.**  (16) $$\left(\hat{\theta },\hat{\Delta }\right)=\arg\max_{\bar{\theta },\Delta }\;P\left[y\;\left|\;{\hat{t}}^{j}\;;\;d,\bar{\theta },\Delta \right.\right]$$

It is important to notice that the ML problem in Equation (16) contains the latent variables Δ; to estimate them, we thus resort to a tailored EM approach [13]. To this aim, define then the auxiliary variables:

(17) $$\begin{array}{c} \Sigma_{1}:=\mathrm{diag}\left(1-{\hat{\Delta }}_{k}\right)\;\\ \Sigma_{2}:=\mathrm{diag}\left({\hat{\Delta }}_{k}\right) \end{array}\;\;\begin{array}{c} {\tilde{y}}_{k}:=y_{k}-d\;\\ \tilde{y}:=y-d𝟙 \end{array}\;\;H:=\left[\begin{array}{c} H_{1}\\ \vdots \\ H_{K} \end{array}\right]=\left[\begin{array}{c} H\left({\hat{t}}_{1}^{j}\right)\\ \vdots \\ H\left({\hat{t}}_{K}^{j}\right) \end{array}\right]$$

with 𝟙 being a vector of *K* ones. The computation of $\left(\hat{\theta },\hat{\Delta }\right)$ is performed through the iteration up to convergence (stopping criteria for EM algorithms are usually based on relative or absolute changes in the parameter estimate or in the value of the log likelihood; see, e.g., [14]; in our implementations, we used the absolute changes in the parameter estimate $\hat{\pi }<\epsilon$; where *ϵ* is a small number $1\times 10^{-5}$ in the implementation) of the following two steps:

**E-step**  (18) $$\begin{array}{c} \delta_{k}=\left(1-\hat{\pi }\right)\;N\left({\tilde{y}}_{k}-H_{k}\hat{\theta }-{\hat{\mu }}_{1},{\hat{\sigma }}_{1}^{2}\right)+\hat{\pi }\;N\left({\tilde{y}}_{k}-H_{k}\hat{\theta }-{\hat{\mu }}_{2},{\hat{\sigma }}_{2}^{2}\right),\;k=1,\ldots ,K\\ {\hat{\Delta }}_{k}=\frac{\hat{\pi }N\left({\tilde{y}}_{k}-H_{k}\hat{\theta }-{\hat{\mu }}_{2},{\hat{\sigma }}_{2}^{2}\right)}{\delta_{k}},\;k=1,\ldots ,K \end{array}$$
**M-step**  (19) $$\begin{array}{cccc} C & = & {\hat{\sigma }}_{1}^{2}\;\Sigma_{1}+{\hat{\sigma }}_{2}^{2}\;\Sigma_{2} & \\ \hat{\theta } & = & {\left(H^{T}C^{-1}H\right)}^{-1}\;\;H^{T}C^{-1}\tilde{y} & \\ {\hat{\mu }}_{i} & = & \frac{{𝟙}^{T}\Sigma_{i}}{{𝟙}^{T}\Sigma_{i}𝟙}\left(\tilde{y}-H\hat{\theta }\right) & \;i=1,2\\ {\hat{\sigma }}_{i}^{2} & = & \frac{{\left(\tilde{y}-H\hat{\theta }\right)}^{T}\Sigma_{i}\left(\tilde{y}-H\hat{\theta }\right)}{{𝟙}^{T}\Sigma_{i}𝟙} & \;i=1,2\\ \hat{\pi } & = & \frac{1}{K}\sum_{k}{\hat{\Delta }}_{k} \end{array}$$

In our numerical experiments, we empirically found it convenient to use the following initial conditions:

(20) $$\hat{\theta }=0,\;{\hat{\mu }}_{1}=0.1,\;{\hat{\mu }}_{2}=-0.1,\;{\hat{\sigma }}_{1}^{2}=0.1,\;{\hat{\sigma }}_{2}^{2}=0.1,\;\hat{\pi }=0.5$$

As for the convergence of the EM to the true ML estimate, we notice that EM algorithms are not in general ensured to have convergence properties. A sufficient condition for convergence is in [15], where the authors show that EM algorithms are convergent if the maximizer of the M-step is unique (a condition that is almost always satisfied in practice). In our case, the M-step maximizer is unique as long as in the update for $\hat{\theta }$ in Equation (19), the matrix $H^{T}C^{-1}H$ admits the inverse. In general, e.g., in both the polynomial case of Example 1 and in the Fourier expansion case of Example 2, this translates into the need for at least *N* samples associated with *N* different laser junction temperatures $t_{k}^{j}$.

## 6. Testing Model Equation (8)

We now devise a numerical algorithm for estimating *d* and the values of the lasing mode selection variables $\Delta_{k}$ starting from the model trained in Section 5 (*i.e.*, an estimated vector $\hat{\bar{\theta }}:={\left[{\hat{\theta }}^{T},\;{\hat{\mu }}_{1},\;{\hat{\mu }}_{2},\;{\hat{\sigma }}_{1}^{2},\;{\hat{\sigma }}_{2}^{2},\;\hat{\pi }\right]}^{T}$ and the statistics of $w_{k}^{1}$, $w_{k}^{2}$ and $\Delta_{k}$) and a set of measurements $y_{k}$ and $t_{k}^{s}$ for k=1,…,K.

Assuming once again to transform the temperature sensor measurements $t^{s}$ into estimated laser junction temperatures ${\hat{t}}^{j}=Wt^{s}$ through Equation (7), the problem of estimating *d* and the $\Delta_{k}$s can be cast as:

(21) $$\left(\hat{d},\hat{\Delta }\right)=\arg\max_{d,\Delta }\;P\left[y\;\left|\;{\hat{t}}^{j}\;;\;d,\hat{\bar{\theta }},\Delta \right.\right]$$

As before, we compute this ML estimate through an EM approach:

**E-step**  (22) $$\begin{array}{c} \delta_{k}=\left(1-\hat{\pi }\right)N\left(y_{k}-\hat{d}-H_{k}\hat{\theta }-{\hat{\mu }}_{1},{\hat{\sigma }}_{1}^{2}\right)+\hat{\pi }N\left(y_{k}-\hat{d}-H_{k}\hat{\theta }-{\hat{\mu }}_{2},{\hat{\sigma }}_{2}^{2}\right),\;k=1,\ldots ,K\\ {\hat{\Delta }}_{k}=\frac{\hat{\pi }N\left(y_{k}-\hat{d}-H_{k}\hat{\theta }-{\hat{\mu }}_{2},{\hat{\sigma }}_{2}^{2}\right)}{\delta_{k}},\;k=1,\ldots ,K \end{array}$$
**M-step**  (23) $$\begin{array}{cccc} C & = & \Sigma_{1}{\hat{\sigma }}_{1}^{2}+\Sigma_{2}{\hat{\sigma }}_{2}^{2} & \\ \hat{d} & = & {\left({𝟙}^{T}C^{-𝟙}𝟙\right)}^{-1}1^{T}C^{-1}\left(y-H\hat{\theta }\right) & \end{array}$$

The same values of $\mu_{1},\mu_{2},\sigma_{1}^{2}$ and $\sigma_{2}^{2}$ at the end of the training step will be used in the testing step. There is no need to recompute them again. In our experiments, we found it beneficial to start from the initial conditions $\hat{d}=\frac{{𝟙}^{T}y}{K}$ and ${\hat{\Delta }}_{k}=0.5$. The convergence properties of this EM procedure are then very similar to the EM in Section 5.

## 7. Designing Model Equation (8)

This section is divided into two parts:

1. Section 7.1, suggesting some hints for designing different structures for $H\left(\cdot \right)$ (e.g., choosing the order for Example 1, for Example 2 or also designing different functional structures depending on the collected information);
2. Section 7.2, suggesting a numerical algorithm for discriminating between different competing structures for *H* starting from data collected in a controlled environment.

### 7.1. Designing $H\left(\cdot \right)$

The proposed EM algorithms have the numerically-favorable property of having both the E and the M steps solvable in closed form. It is important to notice that this is induced by the fact that model Equation (8) is linear in ***θ***, *i.e.*,

(24) $$H\left(t_{k}^{j}\right)\theta =\left[H_{1}\left(t_{k}^{j}\right),\ldots ,H_{N}\left(t_{k}^{j}\right)\right]\left[\begin{array}{c} \theta_{1}\\ \vdots \\ \theta_{N} \end{array}\right]=\sum_{n=1}^{N}H_{n}\left(t_{k}^{j}\right)\theta_{n}$$

Thus, with this structure, the designer can model the effect of the temperature $t_{k}^{j}$ on the measurement $y_{k}$ as the sum of *N* independent effects, each one represented as an opportune generic function of $t_{k}^{j}$ (the weight of which is actually assumed unknown before the training phase).

As shown in Figure 6 and Figure 7, the structures proposed in Examples 1 and 2 have quite general generalization capabilities. Nonetheless, the designer can tailor *H* so that it resembles other structures; our suggestion is to start from raw measured data spanning different temperatures, check visually how the macroscopic temperature trend behaves and then decompose this trend as the sum of different functions that will become the various $H_{n}\left(\cdot \right)$ in model Equation (8).

### 7.2. Determining the Best $H\left(\cdot \right)$ among Different Competing Potential Structures

The process described in Section 7.1 may lead to different competing structures for $H\left(\cdot \right)$. In other words, the designer may propose different structures $H^{\left(1\right)}$, $H^{\left(2\right)}$, *etc.*, and would like to choose the “best” $H^{\left(i\right)}$ given a dataset containing y, $t^{s}$ and the true distance *d*.

We propose to use the classical approach of discriminating the various $H^{\left(i\right)}$’s considering their goodness of fit, *i.e.*, to use GLR [16], for which we first estimate the best estimates ${\hat{\bar{\theta }}}^{\left(i\right)}$ and ${\hat{\Delta }}^{\left(i\right)}$ for each $H^{\left(i\right)}$ given the dataset and then select the best hypothesis considering their resulting log-likelihoods. More formally, the suggested procedure is as in Algorithm 2.

**Algorithm 2** Selection of the best $H^{\left(i\right)}$.
1:
**for** i=1,2,… **do**
2:    Compute ${\hat{\bar{\theta }}}^{\left(i\right)}$ and ${\hat{\Delta }}^{\left(i\right)}$ as in Section 5;3:    Compute
(25)ℓ(i):=+log(σ^12)𝟙TΣ1𝟙+log(σ^22)𝟙TΣ2𝟙+12σ^12y-d𝟙-H(i)θ^(i)-μ^1(i)𝟙TΣ1(i)y-d𝟙-H(i)θ^(i)-μ^1(i)𝟙+12σ^22y-d𝟙-H(i)θ^(i)-μ^2(i)𝟙TΣ2(i)y-d𝟙-H(i)θ^(i)-μ^2(i)𝟙
where $\Sigma_{1}^{\left(i\right)}:=\mathrm{diag}\left(1-{\hat{\Delta }}_{k}^{\left(i\right)}\right)$ and $\Sigma_{2}^{\left(i\right)}:=\mathrm{diag}\left({\hat{\Delta }}_{k}^{\left(i\right)}\right)$;4:
**end for**
5:Select that $H^{\left(i\right)}$ that corresponds to the maximal $\ell^{\left(i\right)}$.


## 8. Experiments

We now validate the proposed thermal model and estimation strategy on real data, aiming to show their effectiveness. We thus consider the simple scenario where a target is fixed in front of a sensor, under constant light and electrical conditions, so that the actual distance between the sensor and the target *d* is fixed. We consider a SICK LMS 200 LiDAR, one of the most widely-used LiDARs in industry and robotics applications.

### 8.1. Training and Validation of the Thermal Model Equation (5)

**Figure 8:**
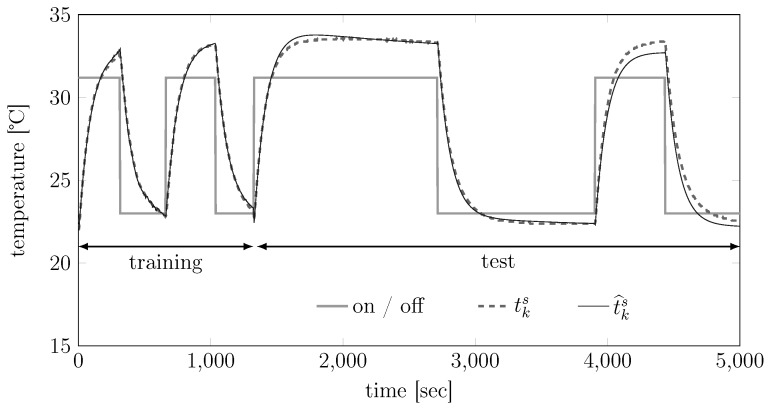
Validation of the thermal model Equation (5) on a SICK 200 LiDAR.

To train and validate the thermal model Equation (5), we conducted the experiment summarized in Figure 8: in a thermally-controlled room at 22°, we performed several on-off cycles of the device and measured the corresponding case temperature $t_{k}^{s}$. We then used the data represented as “training data” in the figure, the data-sheet of the SICK LMS 200 LiDAR and Algorithm 1 to train model Equation (5). After that, we drove the trained model with the ${\bar{u}}_{k}$ in the “test data” as inputs and obtained a predicted temperature ${\hat{y}}_{k}^{s}$. The goodness of fit of the predicted temperatures, computed as:

(26) $$100\left(1-\frac{\sum_{k}{\left({\hat{y}}_{k}^{s}-y_{k}^{s}\right)}^{2}}{\sum_{k}{\left(y_{k}^{s}\right)}^{2}}\right)$$

is then 92.79%. This indicates a very good fit, *i.e.*, a good approximation capability of our proposed thermal model (and associated learning algorithm).

### 8.2. Selection of the Optimal H(·) for the SICK 200 LiDAR

We used Algorithm 2 on real data from a SICK LMS 200 to discriminate between different $H^{\left(i\right)}$’s in Examples 1 and 2, *i.e.*, the hypotheses:

(27) $$H_{i}:y∼\left(1-\Delta \right)N\left(d+H^{\left(i\right)}\theta +\mu_{1},\sigma_{1}^{2}\right)+\Delta N\left(d+H^{\left(i\right)}\theta +\mu_{2},\sigma_{2}^{2}\right)$$

against the null hypothesis:

(28) $$H_{0}:y∼N\left(d,\sigma^{2}\right)$$

so that the log likelihood ratio test between two hypotheses can be defined as:

(29) $$\Lambda_{i}\left(y\right)=\frac{\ell \left(\hat{d},{\hat{\sigma }}^{2}|y\right)}{\ell \left(\hat{d},{\hat{\sigma }}_{1}^{2},{\hat{\sigma }}_{2}^{2},{\hat{\mu }}_{1},{\hat{\mu }}_{2},\hat{\theta },\hat{\Delta }|y,H^{\left(i\right)}\right)}$$

where we explicitly mention the dependence of the log likelihood *ℓ* from the various parameters of the considered model.

**Figure 9:**
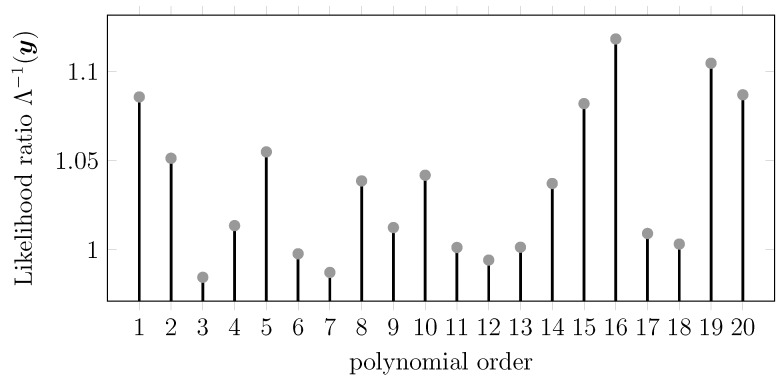
Generalized likelihood ratio (GLR) for Example 10 (polynomial model). The vertical axis values represent the likelihood ratio, and the horizontal axis represents the polynomial order used for generating the $H^{\left(i\right)}$ matrix.

**Figure 10:**
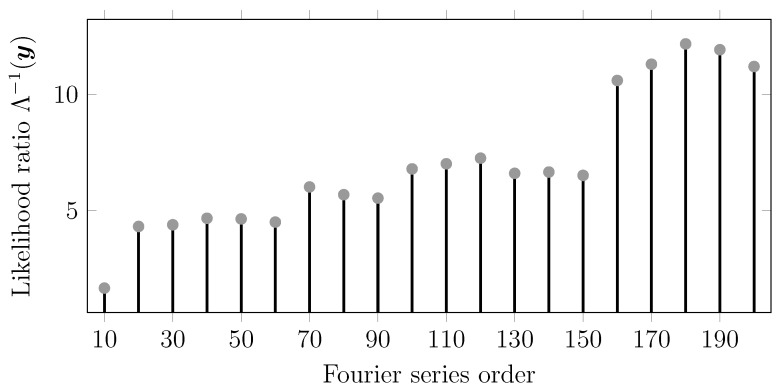
GLR for Example 12 (Fourier model) with different orders. The vertical axis values represent the likelihood ratio and the horizontal axis represents the Fourier series order used for generating the $H^{\left(i\right)}$ matrix.

Figure 9 and Figure 10 show the likelihood ratios obtained for different candidates for polynomial and Fourier expansions, respectively. For the polynomial case, we obtained the highest likelihood for the polynomial order N=16, while for the Fourier case, we obtained the optimal value for N=180. We motivate this latter order, much higher than that of the polynomial case, to be due to the periodic nature of the Fourier model.

### 8.3. Assessment of the Performance Improvement for the SICK 200 LiDAR

**Figure 11:**
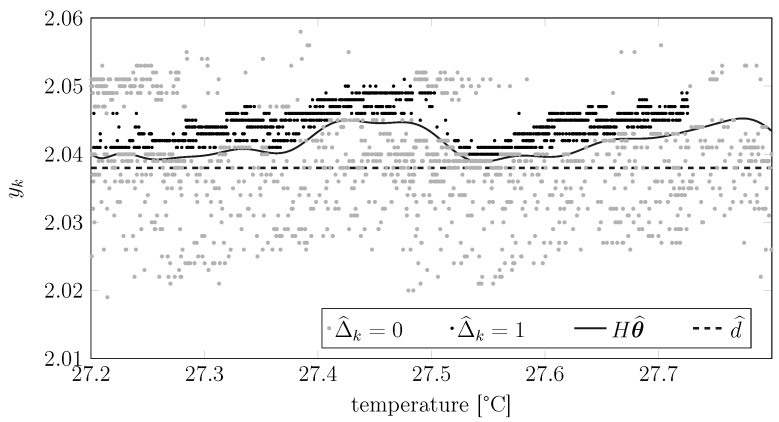
Application of the EM testing algorithm on real data, where the raw measurements are plotted *versus* the temperature of the device. The black dots are the raw measurements that have been associated with lasing Mode 1, while the gray dots are the measurements associated with lasing Mode 2. The solid line denotes the temperature-induced bias on the measured distance.

Assume considering the best model order as in Section 8.2 and applying the main EM estimation algorithm for testing purposes. Figure 11 then shows an example of the outcome of this test on real data: first, the figure plots for each raw measurement the associated lasing modes detected by the EM testing procedure. The same plot shows also the bias induced by the temperature, *i.e.*, $H\hat{\theta }$.

**Figure 12:**
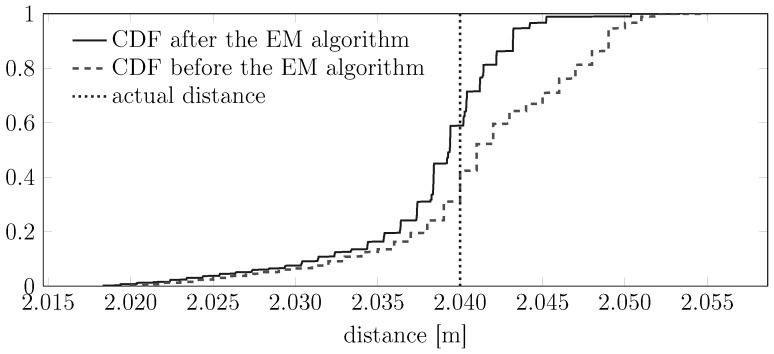
Effects of compensating some raw data with the biases corresponding to the lasing mode of each sample.

Once the raw measurements have been associated with the corresponding lasing modes, it is then possible to subtract from them the corresponding noise bias ${\hat{\mu }}_{1}$ or ${\hat{\mu }}_{2}$; in other words, it is possible to remove a certain lasing mode-dependent bias from each of the various measurements. This leads to a new distribution of the measurements, as shown in Figure 12. This figure shows the cumulative distribution of the measurements that we plotted in Figure 11 before and after subtracting the biases induced by the lasing modes. It is clearly visible that after this removal, the cumulative distribution becomes sharper, indicating that the novel dataset has a smaller variance.

### 8.4. Convergence Properties for the EM Algorithms

**Figure 13:**
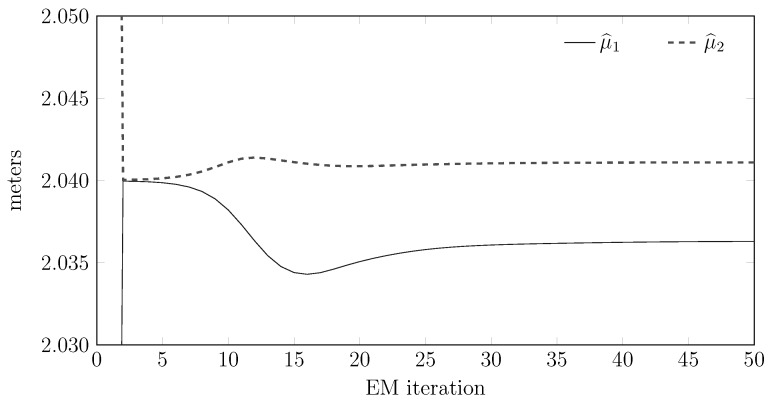
Convergence of the estimated means to their final estimates during the training phase for a training set containing 4000 raw measurements.

**Figure 14:**
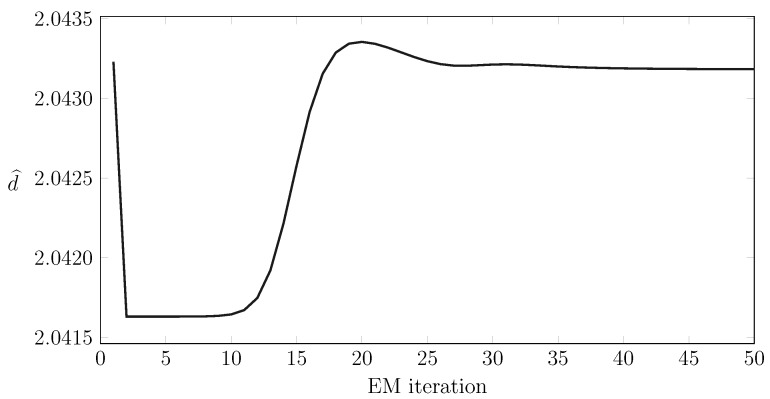
Convergence of the estimated distance to its final estimate during the test phase for a test set containing 4000 raw measurements.

**Figure 15:**
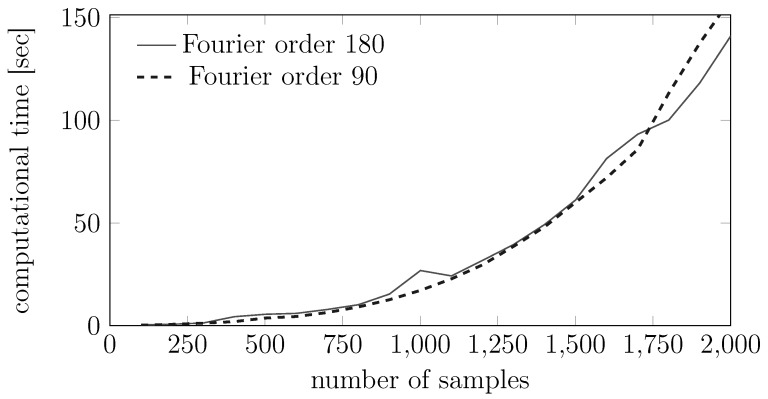
Dependence of the computational complexity for the EM testing phase for two different Fourier order models as a function of the number of samples in the test set.

We now report some numerical results for the convergence properties of the EM training and test algorithms. More precisely, Figure 13 shows the evolution of the estimates during the training phase, while Figure 14 shows the same evolution for the testing phase. We also plot in Figure 15 the computational burden of performing a fixed number of EM test steps for different measurement vector lengths and show empirically how the computational efforts for testing some points are quadratic with the number of samples. Importantly, this computational effort does not depend heavily on the order of the model.

**Figure 16:**
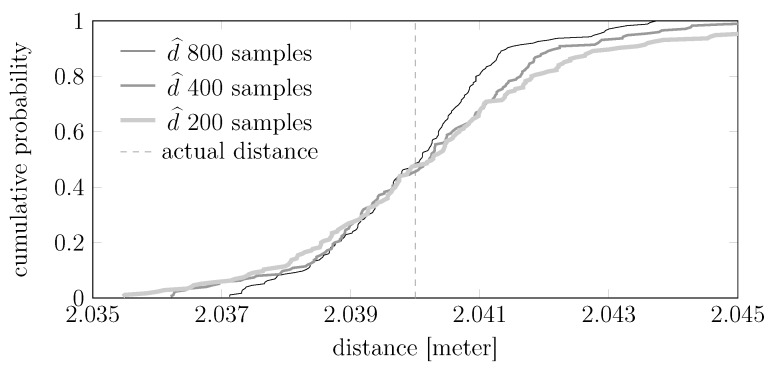
Cumulative distributions of the estimate $\hat{d}$ for different testing sample sizes. The device temperature ranged in these experiments between 24.8 °C and 28.8 °C.

We show in Figure 16 how the precision and accuracy of the estimate $\hat{d}$ changes with the number of samples in the test set. More precisely, we show the following cumulative distribution: given the set of 20,000 raw measurements, extract every subset of 200, 400 or 800 consecutive measurements and, for each of these subsets, compute an estimate $\hat{d}$. The result is intuitive: estimating from more measurements leads to a better estimator (*i.e.*, a cumulative distribution that steepens in correspondence of the true distance).

**Figure 17:**
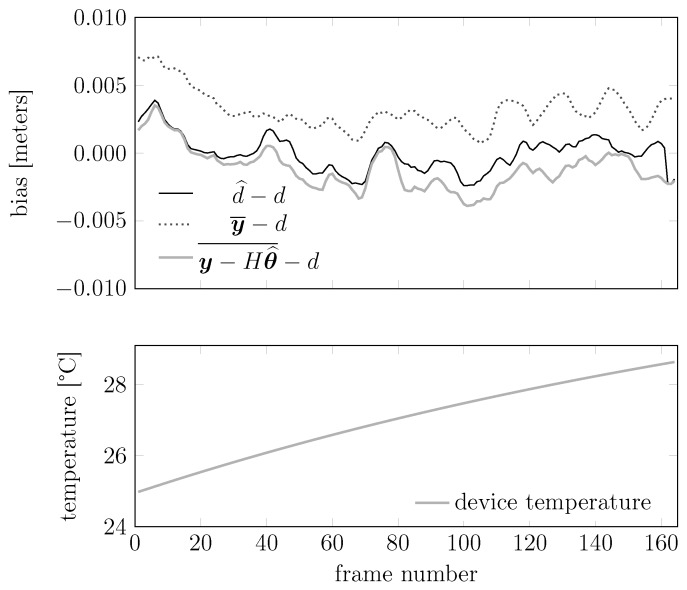
Realizations of the errors committed by the three estimators $\hat{d}$, $\bar{y}$ and $\bar{y-H\theta }$. In this experiment, the temperature of the device ranged between 24.8 °C and 28.8 °C; the sample size is 1000 points.

We show in Figure 17 the realizations of the errors committed by three different estimators: $\hat{d}$ (our estimator), $\bar{y}$ (the sample average of a window of measurements) and $\bar{y-H\hat{\theta }}$ (the sample average of a window of measurements corrected by the temperature compensation term $H\hat{\theta }$). We notice that $\hat{d}$ commits the smallest errors almost everywhere. To integrate the information, we also plot the temperature of the device in the lower plot. This helps with recognizing that $\hat{d}$ has better performance, especially where the effect of the temperature is higher.

**Figure 18:**
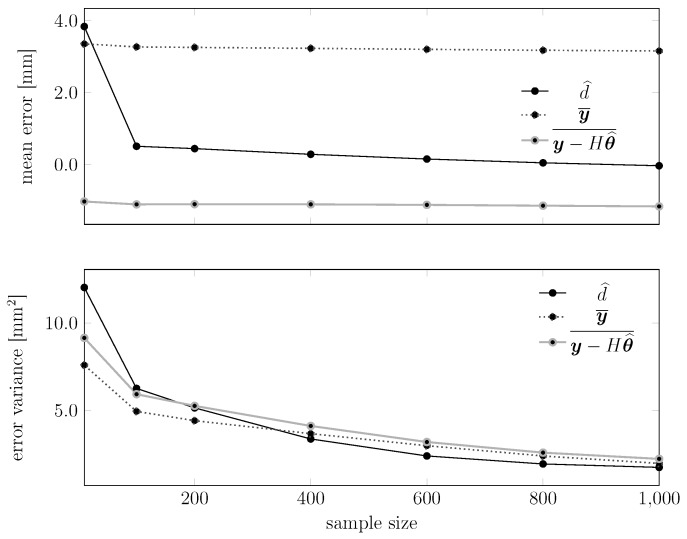
Dependency of the statistical moments of the various estimators on the test sample size. The upper plot shows the mean error, while the lower plot shows the variance of the error. In these experiments, the temperature of the device ranged between 24.8 °C and 28.8 °C.

**Figure 19:**
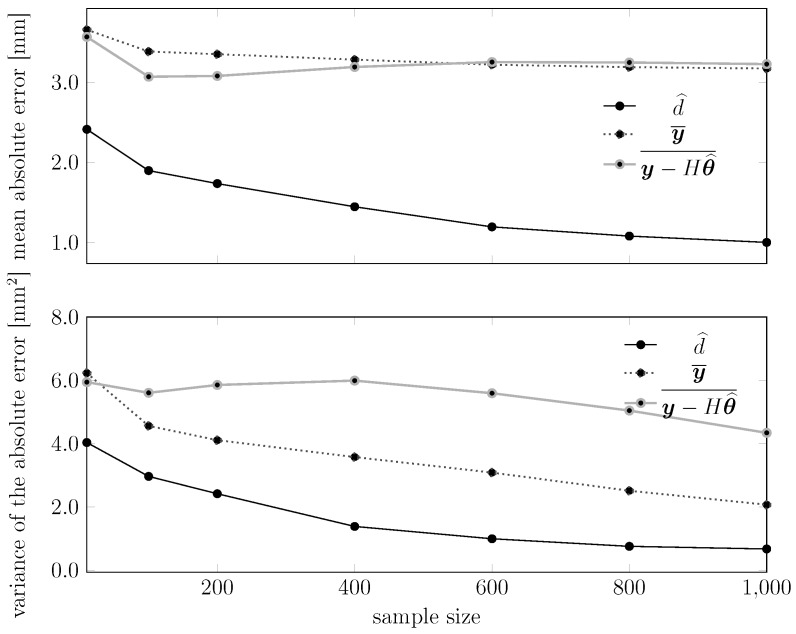
Dependency of the statistical moments of the absolute error of the various estimators on the test sample size. The upper plot shows the mean absolute error, while the lower plot shows the variance of the absolute error. In these experiments, the temperature of the device ranged between 24.8 °C and 28.8 °C.

We also plot in Figure 18 and Figure 19 how the statistical moments of the empirical error and of the empirical absolute error of these estimators behave with the test sample size. Each point in the plot represents the mean or the variance of an error (or absolute error) sequence similar to those shown in Figure 17. We see that the performance of $\hat{d}$ improves monotonically, while for the other estimators, this does not happen. Similar results happen also in the plots for the variances, where the proposed estimator outperforms the other two.

## 9. Conclusions

Physical considerations on the mode-hopping effect lead to the consideration that the measurement noise of time of flight (ToF) laser scanners is intrinsically multi-modal. In its turn, this implies that estimating the actual distance between scanners and the surrounding objects should be performed using latent variable-based statistical models, where the latent variables correspond to the lasing modes of the laser. Since no literature seems to account for this multi-modality, we aimed at closing this gap.

We thus proposed an expectation maximization (EM) algorithm on top of a model that captures biases on the measured distance induced by temperature changes, plus mode-hopping effects through an opportune Gaussian mixture on the measurement noise. Importantly, thanks to a separable model, the EM iterations can be performed analytically. The computational advantages are clear: non-separable models may indeed need to perform the EM iterations numerically, and this would lead to a computational burden hindering the usability of mode-hopping correction procedures in on-line settings.

The proposed strategy incorporates an accurate model of the temperature dynamics of the laser diode. Moreover, to account for the fact that it may be difficult to collect temperature data on the laser, we proposed a strategy for the identification of the parameters of this model that exploit the datasheet of the laser device and some very simple experiments.

Overall, the proposed temperature compensation strategy led to diminishing the spread of the distribution of real measurements from a SICK LMS 200 around the true value *d*, as shown in Figure 12, with corresponding decays of the variance of the absolute error from 2.0 mm${}^{2}$ to 0.68 mm${}^{2}$ and shift of the expected absolute error from 3 mm to 1 mm, as shown in Figure 19.

We eventually notice that the proposed strategy is still in its infancy: indeed, we considered a frequentist case for which the actual distance *d* is a deterministic and fixed quantity, aiming at showing that it is possible to improve the overall precision of a laser scanner through accounting for mode-hopping effects. Nonetheless, in real scenarios, *d* will vary; we thus devise future efforts focusing on strategies for which *d* is a stochastic process that varies according to some given *a priori* distribution.

## Appendix

**Proof of Proposition 1.** By the definition of conditional probability,

(30) $$\begin{array}{cc} P\left[y,{\hat{t}}^{j}\;;\;d,\bar{\theta },\Delta \right]\;\;\;\;\;\; & =P\left[y\;\left|\;{\hat{t}}^{j}\;;\;d,\bar{\theta },\Delta \right.\right]\;P\left[{\hat{t}}^{j}\;;\;d,\bar{\theta },\Delta \right]\\ & =P\left[y\;\left|\;{\hat{t}}^{j}\;;\;d,\bar{\theta },\Delta \right.\right]P\left[{\hat{t}}^{j}\right] \end{array}$$

since ${\hat{t}}^{j}$ does not statistically depend on the (deterministic) parameters *d*, $\bar{\theta }$ and Δ. ☐

**Proof of the structure of the EM training algorithm.** The probability density of y given ${\hat{t}}^{j}$ (and thus *H*) is:

(31) pyt^j;d,θ¯,Δ=11-πNy-d𝟙-Hθ-μ1𝟙,σ12+πNy-d𝟙-Hθ-μ2𝟙,σ22

Knowing the latent variables Δ leads thus to the log-likelihood:

(32) ℓθ¯;y,t^j,d,Δ=1y-d𝟙-Hθ-μ1𝟙TΣ1y-d𝟙-Hθ-μ1𝟙+y-d𝟙-Hθ-μ2𝟙TΣ2y-d𝟙-Hθ-μ2𝟙

Calculating the zero of the score with respect to $\mu_{i}$ and then calculating the zero of the score with respect to ***θ*** leads to the update rules for both $\hat{\mu_{i}}$ and $\hat{\theta_{i}}$. As for the update rule for ${\hat{\sigma }}_{i}^{2}$, we notice that it derives immediately from the fact that the score with respect to $\sigma_{i}$ is:

(33) $$\begin{array}{c} -\frac{K}{\sigma_{i}^{2}}+\frac{1}{\sigma_{i}^{4}}\left({\left(y-d𝟙-H\theta +\mu_{1}𝟙\right)}^{T}\frac{\Sigma_{1}}{K}\left(y-d𝟙-H\theta +\mu_{1}𝟙\right)\right.\;\\ \left.\;+{\left(y-d𝟙-H\theta -\mu_{2}𝟙\right)}^{T}\frac{\Sigma_{2}}{K}\left(y-d𝟙-H\theta -\mu_{2}𝟙\right)\right) \end{array}$$

☐

**Proof of the structure of the EM testing algorithm.** The update rules come directly from the structure of the log-likelihoods in the proof of the structure of the EM training algorithm. ☐
